# Quality Status and Skin-Related Functional Properties of Traditional Korean Fermented Vinegars

**DOI:** 10.3390/foods14152728

**Published:** 2025-08-04

**Authors:** Hwan Hee Yu, So-Won Jang, Eungyeong Kim, Jong-Chan Kim, Mi Jang

**Affiliations:** Food Standard Research Center, Korea Food Research Institute, Wanju 55365, Republic of Korea; yhh@kfri.re.kr (H.H.Y.); wish@kfri.re.kr (S.-W.J.); lpsek@wku.ac.kr (E.K.); jckim@kfri.re.kr (J.-C.K.)

**Keywords:** fermented vinegar, organic acid, amino acid, collagenase inhibition, tyrosinase inhibition

## Abstract

The correlation between fermented vinegar’s physicochemical properties and functional characteristics, particularly skin-related functionalities, remains unclear. We analyzed the quality of widely consumed Korean fermented vinegars, including grain and persimmon vinegars, and their correlation with skin-related functionalities to establish quality control criteria linked to functional properties. Fifteen traditional Korean grain vinegars and fourteen persimmon vinegars were collected; distilled white vinegar was used as the control group. Grain vinegars showed 3.57–100.00% collagenase and 62.38–77.03% tyrosinase inhibition; persimmon vinegars showed 0.00–94.50% and 30.75–71.54%, respectively. To determine which quality characteristics are high in fermented vinegar with high skin-related functionality, a correlation analysis was conducted. In grain vinegar, total nitrogen and free amino acids were strongly associated with skin-related functionalities. In persimmon vinegar, organic acids, particularly lactic acid, were correlated with skin-related effects; thus, both demonstrated the importance of quality assessment. Insights into relationships between the composition and functional properties of fermented vinegar were gained. Specific quality markers for managing skin-related functionality of Korean fermented vinegar established a scientific basis for standardizing quality control, developing high-value functional vinegar products, and ensuring consistent product quality.

## 1. Introduction

Vinegar is a traditional fermented food produced using various raw materials and fermentation methods. It contains bioactive compounds, including organic acids, amino acids, and phenolic compounds, which contribute to its role as a seasoning and preservative as well as its recognition as a functional food [1]. In recent years, interest in functional foods has increased in the food industry, and vinegar has gained attention for its antioxidant, antimicrobial, fat-reducing, and antihypertensive activities [2]. Consequently, studies on the physicochemical properties and bioactive components of fermented vinegars have been actively conducted [3,4,5].

Different types of vinegar are produced globally using unique raw materials and fermentation techniques. In Japan, kurozu (black vinegar) is produced by fermenting brown rice and rice koji over an extended period, resulting in a product known for its umami flavor and high amino acid content [6,7]. In Italy, balsamic vinegar, produced from grapes and aged in oak barrels, is highly regarded for its rich flavor and potent antioxidant properties [8]. In the case of China, over 20 varieties of cereal vinegars are produced. The four well-known and widely consumed types are Shanxi aged vinegar, Zhenjiang aromatic vinegar, Sichuan bran vinegar, and Fujian Monascus vinegar. In addition to these, other types, such as Longmen vinegar and Zhengrong rice vinegar, are also frequently used and remain popular in Chinese cuisine [9]. These examples highlight the diversity of vinegar production across countries, emphasizing the need for comparative studies on the characteristics and functionalities of fermented vinegar. In Korea, traditional vinegar is primarily made from cereals and fruits, with grain and persimmon vinegars being the most widely consumed types [10]. These vinegars are widely used as condiments and functional foods because of their potential health benefits. Notably, the composition and concentration of organic acids, amino acids, and substances related to the antioxidant activity generated during fermentation vary depending on the raw materials and fermentation conditions, necessitating systematic research on their characteristics [11,12]. Given the increasing interest in traditional fermented foods and the global expansion of the functional food market, a systematic evaluation of the quality characteristics of Korean persimmon and grain vinegars is essential to establish scientific standards, ensure product consistency, and support their potential applications as health-promoting ingredients.

Among the various functions of fermented vinegars, several have been reported to have a range of skin-related benefits. Chaff vinegar, which is enriched with active polyphenols, exhibits promising cosmeceutical properties by inhibiting collagenase activity, suggesting its potential for anti-wrinkle and skin-brightening applications [13]. Yun et al. investigated biomarkers related to the anti-aging effect of blended mandarin vinegar and confirmed that it was effective in recovering cell lifespan under acute oxidative stress conditions [14]. Rice vinegar-containing Monascus-fermented soybeans have demonstrated notable cosmeceutical properties, exhibiting potent tyrosinase and elastase inhibitory activities, and substantial antioxidant capacities [15]. Twelve-week intake of a black vinegar drink containing mangosteen pericarp extract reduced skin advanced glycation end products (AGEs) accumulation and improved skin elasticity, demonstrating its anti-glycative and skin-enhancing effects [16]. These studies suggest that vinegars can enhance skin health by providing antioxidant protection, improving skin elasticity, and reducing wrinkles.

Fermentation can increase the bioavailability of active compounds, such as polyphenols, which are believed to contribute to these beneficial effects. Consequently, fermented vinegars are gaining attention as promising natural ingredients for anti-aging and skin-brightening applications. Although various studies have been conducted on the quality and functionality of fermented vinegar in Korea, few have investigated the correlation between its physicochemical properties and functional characteristics, particularly those related to skin. Establishing clear links between the physicochemical quality indicators and skin-related functions is essential for developing high-value functional vinegar products. However, the current Korean fermented vinegar production industry has difficulty selecting quality indicators that can be industrially managed to achieve skin-related functionality.

This study evaluated the physicochemical properties, antioxidant capacities, and enzyme activities related to the skin functionalities of grain and persimmon vinegars, which are among the most widely consumed vinegars in Korea, to derive a functional management strategy through correlation analysis.

## 2. Materials and Methods

### 2.1. Preparation of Korean Fermented Vinegar Samples

To collect products classified as fermented vinegar, we checked the labeling and selected grain vinegar as a product using acetic acid fermentation with alcoholic beverage as the main ingredients using grain source (brown and white rice) and Nuruk (a traditional Korean fermentation starter that cultures microorganisms in grain-based ingredients such as wheat and rice). Persimmon vinegar was selected only as a product fermented with persimmon as the main ingredient. All collected samples were produced in South Korea. Fermented vinegars containing raw materials that could affect other quality factors were excluded. Fifteen grain vinegars and fourteen persimmon vinegars were collected and analyzed for their physicochemical properties, antioxidant activity, and enzyme activity. Distilled white vinegar made from grain-derived ethanol was used as a control for comparison.

### 2.2. Physicochemical Analysis

#### 2.2.1. pH

The pH was measured by immersing the probe of a pH meter (Orion Star A214; Thermo Fisher Scientific, Waltham, MA, USA) in 10 mL of vinegar.

#### 2.2.2. Titratable Acidity

To measure the titratable acidity, we diluted 10 mL of vinegar with water to a final volume of 100 mL. Subsequently, 20 mL of the diluted solution was titrated with 0.1 N NaOH using 1% phenolphthalein as an indicator. The total acidity was calculated using the following formula:

Titratable acidity (%, w/v) = V1 × C × f × 100/V2

where V1 is the volume of NaOH (0.1 N) used for titration (mL), C is the amount of organic acid equivalent to 1 mL of 0.1 N NaOH (acetic acid: 0.006), f is the factor of 0.1 N NaOH, and V2 is the sample volume (mL).

#### 2.2.3. Brix

The Brix of fermented vinegar is the content of available solids (°Brix, 20 °C), which is the value that appears when an appropriate amount is placed on the prism of a refractometer (PR-101α, Atago Co. Ltd., Tokyo, Japan).

#### 2.2.4. Total Nitrogen

Total nitrogen was measured using the method described in the Korea Food Code [17]. Briefly, approximately 1 g of the vinegar sample was accurately measured and placed in a digestion tube, and two digestion-accelerator tablets were added. Next, 12 mL of concentrated sulfuric acid was added to the digestion tube, and the solution was digested for 45–60 min in a digestion device at 420 °C; when the color of the digestion solution changed, it was cooled to room temperature. Then, distilled water (80 mL) was added to the decomposed test solution; further, 25 mL of the collecting solution mixed with the mixed indicator was added to an Erlenmeyer flask, which was placed in the distillation apparatus, and the flask stand was lifted. During distillation, the distillate entered the collecting solution.

Next, 50 mL of 40% NaOH was added to the decomposition tube. They were then distilled in a distillation apparatus for 4 min. The collecting solution in the Erlenmeyer flask of the distillation apparatus turned green as it collected the alkaline substances (such as ammonia) contained in the distillate. The distillate was titrated with hydrochloric acid until the endpoint reached a light pink color. The amount of acid used for titration was recorded.

### 2.3. Free Sugar Content

The free sugar content of the vinegar was analyzed using the quantitative method for sugar content outlined in the Korea Food Code [17]. HPLC (LC-4000, JASCO, Tokyo, Japan), and a refractive index (RI) detector was used to prepare standard fructose, glucose, sucrose, maltose, and lactose solutions. Each standard stock solution was prepared by dissolving 1 g of the standard in 10 mL of water. For the test solution, 5 g of the homogenized sample was weighed into a 50 mL centrifuge tube, followed by the addition of 25 mL of petroleum ether. After centrifugation at 2000 rpm for 10 min, the petroleum ether was removed. This step was repeated, and any residual ether was evaporated using nitrogen. If fat removal was deemed unnecessary, the process was omitted. For sugar extraction, 25 mL of distilled water or 50% ethanol was added to the defatted sample, which was then heated in an 85 °C water bath for 25 min and cooled to room temperature. The weight of the extracted sample was adjusted by adding solvent to match the initial weight, filtered through a 0.45 μm nylon membrane, and centrifuged if turbidity was present. HPLC analysis used an RI detector with a μ-Bondapak Carbohydrate column (300 × 4 mm) and 80% acetonitrile as the solvent. The injection volume was 10 µL, the flow rate was 1 mL/min, and the column temperature was maintained at 30 °C. The total analysis time was 45 min. Peaks were verified by matching retention times. The related chromatogram is shown in Figure S1A. Sugar content (g/100 g) was calculated from the calibration curve using the following formula:

Sugar content (g/100 g) = S × {(a × b)/sample weight (g)} × (100/1000)

where S is the sugar concentration of the test solution (mg/mL), a is the total volume of the test solution (mL), and b is the dilution factor. The concentration range of the calibration curves was adjusted to ensure linearity.

### 2.4. Organic Acid Content

Organic acids in the vinegar were analyzed as previously described [18,19]. The total organic acid content and nine organic acids (oxalic, citric, maleic, malic, malonic, succinic, lactic, fumaric, and acetic acids) were analyzed. The standard solution was prepared at an initial concentration of 10,000 ppm, then diluted to 1000 ppm, and further diluted to 1–100 ppm for the working solution. To prepare the sample solution, 5 g of the homogenized sample was mixed with 20 mL of distilled water, sonicated for 20 min, and centrifuged at 4000× *g*. The supernatant was filtered through a Whatman No. 4 filter paper and diluted 10-fold with water before HPLC analysis. HPLC (LC-4000, JASCO, Tokyo, Japan) was performed using an Aminex HPX-87H column (7.8 mm I.D. × 300 mm, Bio-Rad Laboratories, Boulder, CO, USA) with 10 mM sulfuric acid as the mobile phase for seven organic acids (oxalic, citric, maleic, malic, malonic, succinic, and lactic acids) and 4 mM sulfuric acid for fumaric and acetic acids. The flow rate was 1.0 mL/min, the injection volume was 20 µL, and the column temperature was maintained at 40 °C. The analysis was performed at a wavelength of 254 nm. The related chromatogram is shown in Figure S1B.

### 2.5. Free Amino Acid Content

Free amino acids in the fermented vinegar were analyzed using an HPLC (LC-4000, JASCO, Tokyo, Japan) and fluorescence (FLD) detector according to Bhat et al. [20] and Dai et al. [21] with modifications. An amino acid standard mixture (WAT088122, Waters Corporation, Milford, MA, USA) containing alanine, arginine, aspartic acid, cysteine, glutamic acid, glycine, histidine, isoleucine, leucine, lysine, methionine, phenylalanine, proline, serine, threonine, tyrosine, and valine was used as the standard. The reagents utilized in this study are enumerated as follows: ISSS (Internal Standard Stock Solution, 2.5 mM α-aminobutyric acid), ISS (prepared by diluting 20 µL of ISSS with 980 µL of 20 mM HCl), CS/IS (40 µL ISSS + 40 µL CS + 920 µL water), and Standard preparation (40 µL Amino Acid STD + 960 µL water). Sample pretreatment was performed using the AccQ-Flour Reagent. After preheating a block to 55 °C, one vial of AccQ-Flour Reagent powder (2A) and 1 mL of 2B were mixed, vortexed for 10 s, and heated for less than 10 min to dissolve. Derivatization was conducted by mixing 80 µL of AccQ-Flour Borate Buffer with 20 µL of AccQ-Flour Reagent. The standard material was prepared by combining 10 µL of CS/IS, 70 µL of AccQ-Flour Borate Buffer, and 20 µL of AccQ-Flour Reagent, followed by a 1 min room temperature reaction and heating at 55 °C for 10 min. The vinegar sample was prepared similarly by mixing 10 µL of ISS, 20 µL of AccQ-Flour Borate Buffer, and 20 µL of AccQ-Flour Reagent. HPLC analysis was conducted using an FLD detector set at excitation and emission wavelengths of 250 and 395 nm, respectively. A Waters AccQ-Tag Column (3.9 mm × 150 mm, 4 µm) was used. The mobile phase consisted of A (100 mL acetate-phosphate buffer + 900 mL water) and B (acetonitrile). The gradient elution program was initiated with 100% A, followed by a gradual increase of eluent B according to the following schedule: 0 min, 0% B; 0.5 min, 1% B; 18 min, 5% B; 19 min, 9% B; 27 min, 14% B; 43 min, 35% B; 46 min, 0% B; and 50 min, 0% B. The flow rate was 1 mL/min, the injection volume was 10 µL, the column temperature was maintained at 37 °C, and the total analysis time was 45 min. The related chromatogram is shown in Figure S1C.

### 2.6. Antioxidant Capacity

#### 2.6.1. Total Polyphenol Content

Total polyphenol content (TPC) was determined using a method of Ferreira-Santos et al. [22]. To determine the total polyphenol content, 5 µL of the vinegar sample was mixed with 15 µL of 7.5% Folin–Ciocalteu reagent and 170 µL of distilled water. After allowing the mixture to stand for 5 min at room temperature, 60 µL of 7.5% Na_2_CO_3_ solution was added. The reaction was carried out for 1 h at ambient temperature, and the absorbance was subsequently recorded at 750 nm. The TPC was expressed as milligrams of gallic acid equivalents per liter (mg GAE/L), based on a calibration curve prepared with gallic acid standards.

#### 2.6.2. Total Flavonoid Content

Total flavonoid content (TFC) was assessed according to Ochieng et al. [23]. Specifically, 20 µL of the vinegar sample was mixed with 10 µL of 5% NaNO_2_ solution and 80 µL of distilled water. After allowing the mixture to stand for 5 min, 10 µL of 10% AlCl_3_ and 80 µL of 1 M NaOH were sequentially added. The resulting solution was incubated at room temperature for 30 min. Absorbance was measured at 510 nm, and TFC was expressed as milligrams of catechin equivalents per kilogram (mg CE/kg) based on a catechin standard curve.

#### 2.6.3. DPPH (1,1-Diphenyl-2-Picrylhydrazyl) Assay

DPPH radical scavenging activity was measured using some modification of Ballesteros et al. [24]. DPPH radical scavenging activity was determined by adding 190 µL of 0.15 mM DPPH solution to 10 µL of the vinegar sample, incubating in the dark for 30 min, and measuring absorbance at 517 nm. The DPPH radical scavenging activity was expressed as the inhibition concentration at 50% (IC_50_) value (mg TE/L), calculated based on a Trolox calibration curve.

#### 2.6.4. ABTS [2,2′-Azinobis(3-Ethylbenzothiazoline-6-Sulphonic Acid)] Assay

ABTS radical scavenging activity was performed as modification of Abuduaini et al. [25]. ABTS radical scavenging activity was determined by adding 180 µL of ABTS solution (7 mM ABTS + 2.45 mM potassium persulfate) to 20 µL of the sample, incubating in the dark for 30 min, and measuring absorbance at 734 nm. The ABTS radical scavenging activity was calculated as IC_50_ value (mg TE/L) using a Trolox calibration curve.

#### 2.6.5. FRAP (Ferric Reducing Antioxidant Power) Assay

FRAP assay was conducted as described by Meneses et al. [26]. The FRAP assay was performed by adding 290 µL of a solution (10 mM TPTZ/20 mM FeCl_3_/0.3 M acetate buffer = 1:1:10) to 10 µL of the vinegar sample, incubating at 37 °C for 15 min, and measuring absorbance at 593 nm. FRAP assay was calculated as Fe^2+^ equivalents (mmol Fe^2+^/L) using a FeSO_4_ calibration curve.

### 2.7. Enzyme Activity Assay

#### 2.7.1. Collagenase Inhibitory Activity

The buffer for collagenase inhibitory activity was prepared by dissolving 0.1 M Trizma in distilled water, adjusting the pH to 7.5 with HCl, and adding 4 mM calcium chloride. The sample was diluted to 0.1% using a buffer. Substrate (0.5 mg/mL 4-Phenylazobenzyloxycarbonyl-Pro-Leu-Gly-Pro-D-Arg trifluoroacetate salt) and *Clostridium histolyticum* collagenase (0.5 mg/mL) were prepared. In each well of a 96-well plate, 20 µL of sample, 50 µL of substrate, and 30 µL of collagenase were sequentially added. The plates were incubated at 37 °C for 45 min. After the reaction, 100 µL of 6% citric acid was added to stop the reaction, and absorbance was measured at 324 nm. The negative control (NC) was prepared by adding only the substrate without the enzyme, while the positive control (PC) consisted of the substrate and the enzyme without the sample. The experimental group included substrate, enzyme, and the vinegar sample. The collagenase inhibitory activity was calculated using the following formula:

Collagenase inhibitory activity (%) = (1 − Abs of sample/Abs of PC) × 100

#### 2.7.2. Tyrosinase Inhibitory Activity

Tyrosinase inhibitory activity was determined by diluting the sample to 0.0016% with water and preparing 0.02 mg/mL tyrosinase from mushrooms (Sigma-Aldrich, St. Louis, MO, US) in water. In each well of a 96-well plate, 20 µL of diluted sample and 20 µL of tyrosinase solution were added and reacted in a shaker for 10 min at room temperature in the dark. Then, 40 µL of 1 mM L-DOPA was added, and the mixture was reacted for an additional 30 min at room temperature. The absorbance was measured at 475 nm. The NC was prepared by adding only the substrate without the enzyme, while the PC consisted of the substrate and the enzyme without the sample. The experimental group included substrate, enzyme, and the vinegar sample. The tyrosinase inhibitory activity was calculated using the following formula:

Tyrosinase inhibitory activity (%) = (1 − Abs of sample/Abs of PC) × 100

### 2.8. Statistical Analysis

Pearson correlation analysis was performed to analyze the relationship between the variables, and the Pearson correlation coefficient and *p*-value were calculated for each pair of variables. The significance level of *p* < 0.05 was used. The results of the correlation analysis were visualized as a heat map, and variable pairs with significant correlations were highlighted using circular markers. Statistical analyses were performed using the Python SciPy (1.16.0) and Seaborn packages (0.13.2).

## 3. Results and Discussion

### 3.1. Physicochemical Analysis of Korean Fermented Vinegar

**Figure 1:**
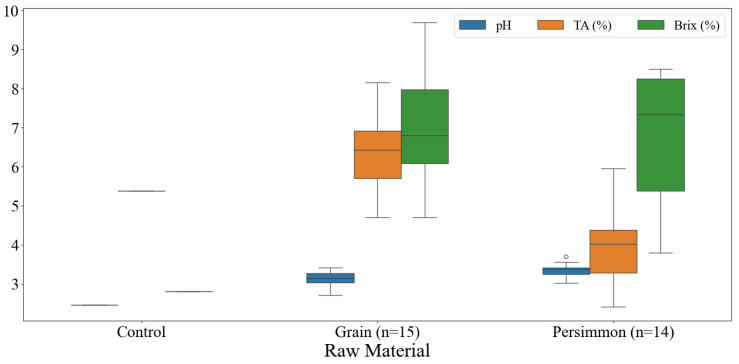
Box plots of physicochemical properties of commercially available fermented vinegars in Korea. TA, titratable acidity.

The results of the physicochemical analysis of traditional Korean grain and persimmon vinegars are shown in Figure 1. The pH of the distilled white vinegar used as a control was 2.46. The pH of the grain vinegar ranged from 2.72 to 3.41, and the pH of the persimmon vinegar was 3.02 to 3.70. The pH of the persimmon vinegar tended to be higher than that of the grain vinegars. The low pH of fermented vinegar results from acetic acid production via alcohol oxidation by acetic acid bacteria during fermentation. Cosmulescu et al. reported that the pH of various fruit vinegars, including apple, wild pear, red hawthorn, cherry, and apple, ranged from 2.32 (sour cherry) to 3.67 (red hawthorn) [27].

According to the Korea Food Code, fermented vinegar must have a total acid content of at least 4%, with persimmon vinegar being the only exception with a content of at least 2.6% [17]. The total acidity of the distilled white vinegar used as the control was 5.38%. The total acidity of grain vinegar ranged from 4.70 to 9.70%, and that of persimmon vinegar was from 2.42 to 5.96%. Among the analyzed Korean fermented vinegar products, only persimmon vinegar did not meet the standards.

Brix is an important indicator of sugar concentration in raw materials, and its value changes during the fermentation process as sugar is converted to alcohol and acetic acid. The Brix value of the distilled white vinegar used as a control was 2.80%. The Brix of grain vinegar ranged from 4.70 to 9.70%, and that of persimmon vinegar was from 3.80 to 8.50%. Brix can be used to determine the sensory characteristics of vinegar or as an indicator of the fermentation process. Ozturk et al. reported a Brix percentage range of 1.22–20.80% for grape vinegar, 1.02–12.90% for apple vinegar, and 1.26% for hawthorn vinegar [28].

### 3.2. Free Sugar Contents of Korean Fermented Vinegar

**Figure 2:**
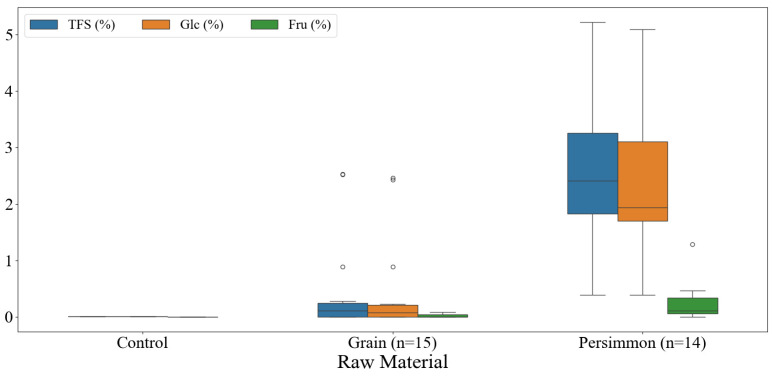
Box plots of sugar contents of commercially available fermented vinegars in Korea. TFS, total free sugar; Glc, glucose; Fru, fructose.

The free sugar contents of traditional Korean grain and persimmon vinegars are shown in Figure 2. The free sugar content of distilled white vinegar was as negligible (0.01%) as that of glucose, and fructose was not detected. The average free sugar content of grain vinegar was 0.47% (0.00–2.53%), of which glucose averaged 0.45% (0.00–2.46%) and 0.02% (0.00–0.09%). Maltose and sucrose were not detected. Most residual free sugar in grain vinegar is glucose derived from grain starch. In the case of persimmon vinegar, the free sugar content averaged 2.62% (0.39–5.22%), glucose content averaged 2.39% (0.39–5.09%), and fructose content averaged 0.23% (0.00–1.29%). Similar to grain vinegar, maltose and sucrose were not detected in the persimmon vinegar.

### 3.3. Organic Acid Contents of Korean Fermented Vinegar

**Figure 3:**
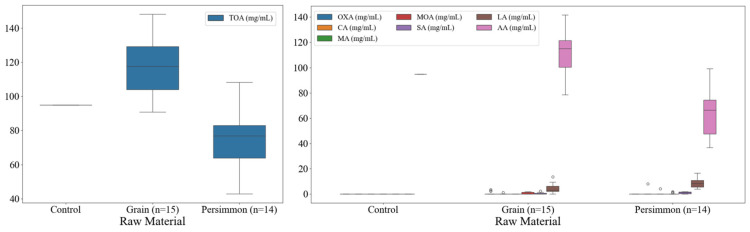
Box plots of organic acid contents of commercially available fermented vinegars in Korea. TOA, total organic acid; OXA, oxalic acid; CA, citric acid; MA, malic acid; MOA, malonic acid; SA, succinic acid; LA, lactic acid; AA, acetic acid.

The organic acid contents of traditional Korean grain and persimmon vinegars are shown in Figure 3. Total organic acid content in the control group was 94.91 mg/mL. The total organic acid content in grain vinegar averaged 117.27 (90.79–148.22 mg/mL). In the case of persimmon vinegar, the average was 75.73 (42.82–108.32 mg/mL), which was lower than that of grain vinegar. Oxalic acid was specifically detected in some grain vinegars, whereas malic acid was present in persimmon vinegar. In addition, citric acid, malonic acid, succinic acid, and lactic acid were detected. Lactic acid was the second most abundant acid in grain and persimmon vinegars, followed by acetic acid, and it tended to be higher in persimmon vinegar than grain vinegar. In the case of acetic acid, which provides the unique taste and flavor of fermented vinegar, grain vinegar showed a higher average of 110.99 mg/mL (78.58–141.71 mg/mL) than persimmon vinegar at 64.92 mg/mL (36.78–99.2 mg/mL).

The key mechanism for producing fermented vinegar is known to be the oxidation of ethanol into acetic acid by acetic acid bacteria. Acetic acid bacteria are Gram-negative or Gram-positive obligate aerobes belonging to the family *Acetobacteraceae*, which are characterized by non-spore-forming, ellipsoidal to rod-shaped cells. They belong to the acetous group of *Acetobacteraceae*, which includes key genera such as *Acetobacter*, *Gluconobacter*, *Gluconacetobacter*, and *Komagataeibacter* [29]. The acetic acid produced by these bacteria is the main component of fermented vinegar.

Lactic acid bacteria, along with yeast and acetic acid bacteria, are important microorganisms involved in the production of naturally fermented vinegar and are more likely to be involved in fruit vinegars that already contain more monosaccharides than grain vinegars, which require an additional saccharification step [30]. This is consistent with our results, which showed that the lactic acid content was higher in persimmon vinegar than in grain vinegar. In addition, lactic acid bacteria are involved in the fermentation of fermented vinegar, and the mutualistic and symbiotic actions of yeast and lactic acid bacteria in alcohol fermentation have been studied to some extent [31,32]. They were found to be present in the early stages of acetic acid fermentation and to affect the quality of vinegar [33].

Although the involvement of lactic acid bacteria and acetic acid bacteria that change during fermentation is very important, this study focused on the physicochemical quality characteristics and functional indicators of commercial fermented vinegar. Such functional characteristics may reflect the underlying contribution of specific microorganisms to skin-related bioactivities. Exopolysaccharides from *Lactobacillus casei* significantly inhibited collagenase and elastase activities in human fibroblasts while also promoting wound healing and downregulating matrix metalloproteinases gene expression [34]. Additionally, it has been reported that Buni cider fermented with a single strain of *Acetobacter xylinum* exhibited high collagen inhibition activity [35]. These findings suggest that microbial cells or their metabolites involved in the fermentation process may contribute to the skin-related bioactivities observed in functional vinegars.

### 3.4. Total Nitrogen and Amino Acid Contents of Korean Fermented Vinegar

**Figure 4:**
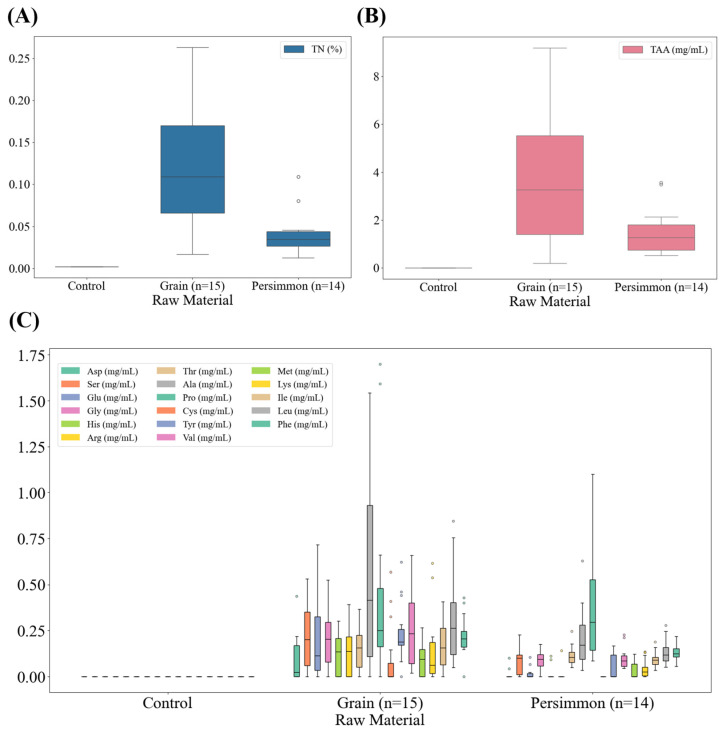
Box plots of total nitrogen and amino acid contents of commercially available fermented vinegars in Korea. (**A**) Box plot of total nitrogen of grain and persimmon vinegar; (**B**) box plot of total amino acid of grain and persimmon vinegar; (**C**) box plot of free amino acid of grain and persimmon vinegar. TN, total nitrogen; TAA, total amino acid; Asp, aspartic acid; Ser, serine; Glu, glutamic acid; Gly, glycine; His, histidine; Arg, arginine; Thr, threonine; Ala, alanine; Pro, proline; Cys, cysteine; Tyr, tyrosine; Val, valine; Met, methionine; Lys, lysine; Ile, isoleucine; Leu, leucine; Phe, phenylalanine.

The total nitrogen and free amino acid contents of traditional Korean grain and persimmon vinegars are shown in Figure 4. The total nitrogen content of distilled white vinegar was 0.0%, whereas that of grain vinegar ranged from 0.02 to 0.26%, and persimmon vinegar demonstrated comparatively low levels of total nitrogen (0.01–0.11%). The nitrogen content of fermented vinegar is derived from proteins in starch-rich substrates such as brown rice. It serves as a quality control indicator, with specific amounts of raw grain materials and fermentation and maturation periods as crucial factors.

The analysis of the total amount of free amino acids in grain vinegars exhibited an average of 3.69 mg/mL (0.19–9.20 mg/mL). In the case of persimmon vinegars, the average was 1.51 mg/mL (0.52–3.56 mg/mL). Amino acids were not detected in distilled white vinegar. The content of each free amino acid showed that grain vinegar contained more types of amino acids and greater amounts than persimmon vinegar; in particular, aspartic acid, glutamic acid, histidine, and arginine were present in higher quantities than in persimmon vinegar. Persimmon vinegar contains a large amount of proline among its free amino acids.

The elevated levels of free amino acids observed in grain vinegar compared to persimmon vinegar could be attributed to the protein in brown rice, the main raw material of grain vinegar, which undergoes breakdown during fermentation. Brown rice undergoes less polishing than white rice, retaining more germ cells and other constituents. Additionally, it contains higher concentrations of proteins and amino acids than white rice [36]. Cereals contain grain-specific proteins such as glutelin and prolamin, which are broken down by enzymes during fermentation. Molds such as *Aspergillus oryzae* and bacteria of the genus *Bacillus* contained in Nuruk (Korean traditional fermentation starter) actively produce starch-decomposing enzymes as well as protein-decomposing enzymes [37].

These enzymes can hydrolyze the starch of brown rice and the protein during the fermentation process. Consequently, amino acids derived from the proteins in the raw materials accumulate in grain vinegar. This is considered the most important factor contributing to the elevated levels of free amino acids found in grain vinegar. The Maillard reaction, a non-enzymatic browning reaction, is responsible for the color change from yellow to dark brown in grain-fermented vinegar because of its relatively high carbohydrate and protein content [38]. This reaction occurs during the aging process, resulting in a change in flavor owing to the production of various compounds, including melanoidin.

### 3.5. Antioxidant Capacities of Korean Fermented Vinegar

**Figure 5:**
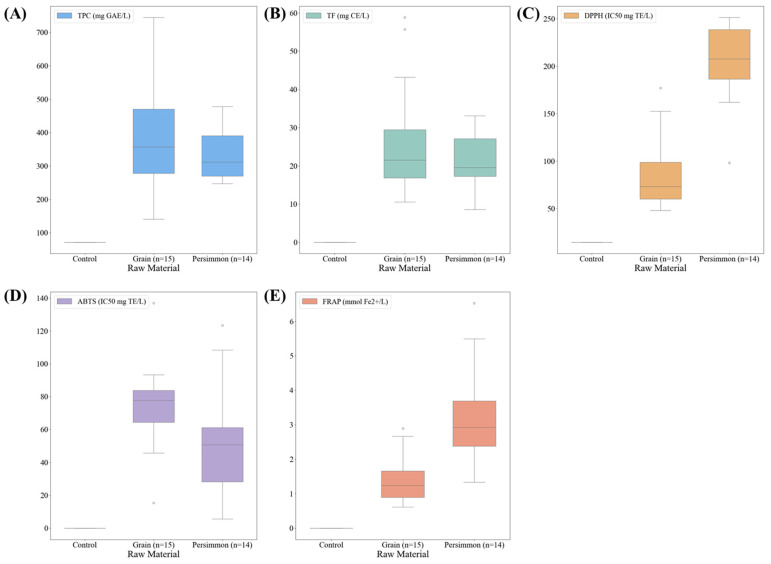
Box plots of antioxidant capacities of commercially available fermented vinegars in Korea. (**A**) Box plot of TPC in grain and persimmon vinegar; (**B**) box plot of TF in grain and persimmon vinegar; (**C**) box plot of DPPH radical scavenging activity in grain and persimmon vinegar; (**D**) box plot of ABTS radical scavenging activity in grain and persimmon vinegar; (**E**) box plot of FRAP in grain and persimmon vinegar. TPC, total polyphenol content; TF, total flavonoid content.

The antioxidant capacities of traditional Korean grain and persimmon vinegars are shown in Figure 5. The average TPC, TF, DPPH, ABTS, and FRAP contents of grain vinegar were 376.49 mg GAE/L (140.43–744.85 mg GAE/L), 26.61 mg CE/L (10.56–58.84), 88.49 IC_50_ mg TE/L (48.12–177.0 IC_50_ mg TE/L), 74.47 IC_50_ mg TE/L (15.31–136.98 IC_50_ mg TE/L), and 1.41 mmol Fe^2+^/L (0.61–2.89 mmol Fe^2+^/L), respectively. Persimmon vinegar showed antioxidant activity of 338.06 mg GAE/L (247.16–478.07 mg GAE/L), 21.23 mg CE/L (8.56–33.14 mg CE/L), 204.68 IC_50_ mg TE/L (98.39–251.47 IC_50_ mg TE/L), 52.96 IC_50_ mg TE/L (5.54–123.40 IC_50_ mg TE/L), and 3.19 mmol Fe^2+^/L (1.33–6.53 mmol Fe^2+^/L). The antioxidant capacities of grain and persimmon vinegars were different, which seems to be because of the antioxidant-related components included, depending on the raw materials and fermentation method. Grain vinegar turns black because of the formation of melanoidins by the Maillard reaction during maturation. These melanoidins significantly affect antioxidant activity [39]. In addition, the high antioxidant activity of grain vinegar is caused by phenolic compounds, and there is a synergistic effect on the antioxidant activity in the presence of organic acids and free amino acids in vinegar [40]. According to Liu et al., the high FRAP and TPC of fruit vinegars can be attributed to organic acids such as tartaric acid, malic acid, lactic acid, citric acid, and succinic acid and to phenolic compounds such as gallic acid, chlorogenic acid, and caffeic acid [41]. According to Sakanaka and Ishihara, persimmon vinegar has a higher antioxidant activity than apple cider vinegar, polished rice vinegar, and unpolished rice vinegar [42].

### 3.6. Skin-Related Functionalities of Korean Fermented Vinegar and Correlation with Qualities of Vinegar

**Figure 6:**
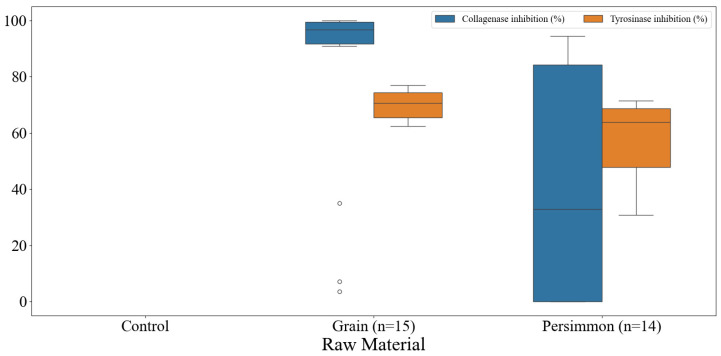
Box plots of collagenase and tyrosinase inhibition effects of commercially available fermented vinegars in Korea.

The results of collagenase and tyrosinase inhibition by traditional Korean grain and persimmon vinegars are shown in Figure 6. Some grain and persimmon vinegars have been found to have strong collagenase and tyrosinase inhibitory effects. In the case of grain vinegars, the collagenase inhibition activity was 3.57–100.00%, and the tyrosinase inhibition activity was 62.38–77.03%. Persimmon vinegar showed collagenase inhibition activity of 0.00–94.50%,] and tyrosinase inhibition activity of 30.75–71.54%. Among the fermented vinegars we collected, some showed high collagenase inhibition and tyrosinase inhibition activities, whereas some did not show such properties. Therefore, we analyzed the correlation between various quality indicators and examined them primarily in relation to skin-related functionality.

**Figure 7:**
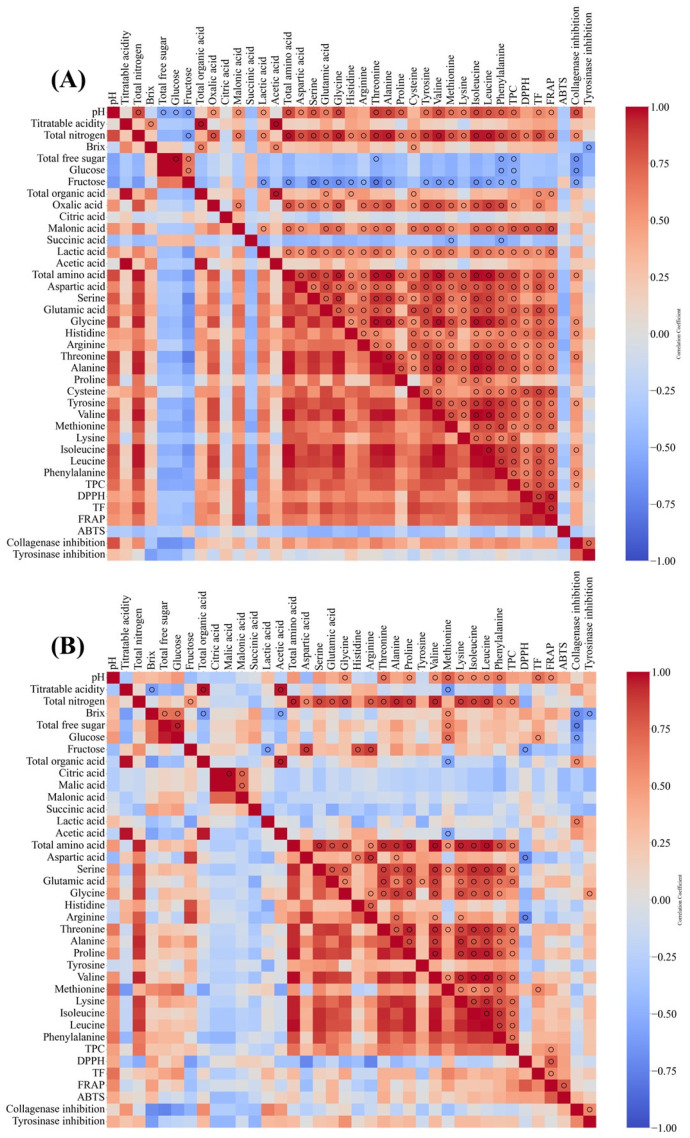
Correlation analysis of quality parameters, antioxidant capacities, and skin-related functionalities of commercially available fermented vinegars in Korea. (**A**) Correlation of various parameters in grain vinegar; (**B**) correlation of various parameters in persimmon vinegar. Statistically significant correlations (*p* < 0.05) are indicated with a circle.

Based on the various analysis results of the fermented vinegar, correlations by indicator were analyzed, and statistically significant relationships are indicated by circles (*p* < 0.05). Figure 7 shows the results of the correlation analysis of various parameters of grain and persimmon vinegars. Notable correlations in grain vinegar revealed that total nitrogen, free amino acids, and antioxidant activity were positively correlated with collagenase inhibition, whereas free sugar levels were negatively correlated. According to Yeerong et al., edible insect extracts with high protein content and antioxidant activity also exhibit high collagenase inhibitory activity, suggesting that protein and antioxidant activity may have influenced this effect [43].

Tyrosinase inhibition activity negatively correlated with Brix expression and positively correlated with collagenase inhibition. In addition, pH significantly affected the antioxidant activity and collagenase inhibition activity, which is thought to be because of the relatively high pH resulting from the content of various amino acids and other nitrogen sources. Persimmon vinegar showed a trend that was different from that of grain vinegar. In persimmon vinegar, total and lactic acid correlated positively with collagenase inhibition, whereas sugar-related indicators (Brix, TFS, and glucose) were negatively correlated. There is insufficient evidence to suggest that lactic acid inhibits collagenase activity. However, this could be indirect evidence of an increase in metabolites that inhibit collagenase, owing to the growth of lactic acid bacteria during vinegar manufacturing. According to Kim et al., lactic acid bacteria cultures exhibited an average collagenase inhibition effect of 23.80%, suggesting that lactic acid and the metabolites produced by lactic acid bacteria may influence collagenase inhibition [44].

The physicochemical parameters of vinegar, such as pH, total acidity, and Brix, are not only key indicators of fermentation completeness but may also influence its biological activity. For instance, lower pH and higher titratable acidity typically reflect the abundance of organic acids, which are known to contribute to antimicrobial and antioxidant activities. Similarly, Brix values, which reflect residual sugars, could indirectly relate to fermentation progress and the potential presence of functional metabolites. Thus, these basic quality parameters could serve as foundational indicators of the functional potential of fermented vinegars.

In our study, no significant correlation was found between various antioxidant activities, collagenase inhibition, and tyrosinase inhibition. However, as many studies have verified the relationship between antioxidant capacity and skin-related functionality, it is necessary to consider issues such as the number of samples and variables in the manufacturing processes [45,46,47]. In particular, the absence of comprehensive metabolite profiling may have limited the identification of bioactive compounds responsible for skin-related functionality. Further studies are needed to identify other potential bioactive compounds that may influence skin-related functionality.

Although it is essential to evaluate skin-related functionality based on antioxidant capacity, evaluating skin-related functionality through physicochemical factors can also be one of the various evaluation methods. Our correlation analysis showed that specific components, such as free amino acids in grain vinegars and organic acids in persimmon vinegars, were positively associated with collagenase and tyrosinase inhibition activities. This is consistent with previous findings that amino acid-rich rice vinegars could enhance collagen protection and anti-wrinkle effects [13,48], while organic acids and phenolic compounds in fruit vinegars contribute to skin-brightening potential [14,35]. These results suggest that managing these quality indicators may help produce fermented vinegars with targeted skin-related functions.

Overall, when skin-related functionality was set as the main management point for manufacturing traditional Korean fermented vinegar, it was concluded that grain vinegar should focus on managing free amino acids, and persimmon vinegar should focus on managing organic acids. In addition, as the conversion of sugars derived from starch or fruits into alcohol and various metabolites is considered important for skin-related functionality, the completeness of the overall fermentation should be confirmed. Although this study identified potential associations between skin-related enzyme inhibitory activities and the physicochemical and compositional characteristics of traditional Korean fermented vinegars, it should be noted that considerable variation was observed among the samples, even when similar raw materials and traditional fermentation methods were used. Since the collected products were manufactured independently by different producers, it is challenging to determine the precise compounds or mechanisms responsible for the observed bioactivities. Therefore, while the present findings may contribute to establishing preliminary quality indicators related to functional potential, further studies are needed to clarify the specific bioactive compounds and the underlying biological mechanisms involved.

## 4. Conclusions

In this study, grain vinegar and persimmon vinegar, traditional Korean fermented vinegars, were collected, and their physicochemical properties, antioxidant activity, and skin-related enzyme-based functionality were studied. The quality status of the Korean fermented vinegars was confirmed by analyzing physicochemical properties, such as pH, titratable acidity, and Brix, as well as total free sugars, organic acids, and free amino acids. Five antioxidant activities (TPC, TF, DPPH, ABTS, and FRAP) of each fermented vinegar were analyzed to compare the antioxidant activities of grain vinegar and persimmon vinegar.

As the same raw materials exhibited different skin-related functionalities, total nitrogen and free amino acids were selected for grain vinegar, whereas total organic acids and lactic acid were selected for persimmon vinegar as indicators for managing skin-related activities. In addition, both vinegars exhibited a negative correlation with the residual sugar, indicating the importance of setting the fermentation endpoint. Therefore, we inferred a correlation that can be used to determine skin-related functionality using physicochemical indices.

Taken together, these findings highlight that managing specific physicochemical markers, such as free amino acids and organic acids, could serve as practical quality indicators for producing fermented vinegars with enhanced skin-related functionality. This provides a scientific basis for manufacturers aiming to develop premium vinegar products targeting cosmeceutical benefits. Despite these findings, this study has several limitations. the microbial communities were not analyzed in the fermented vinegar samples; thus, the roles of specific microbial strains in contributing to functional properties remain speculative. Moreover, the enzyme inhibition results obtained from in vitro assays require further validation using skin cell models or human clinical studies to confirm the actual efficacy in cosmetic applications. These limitations highlight the need for future studies combining microbiological and clinical approaches to fully elucidate the skin-related functionalities of fermented vinegars.

## Data Availability

The original contributions presented in this study are included in the article material/Supplementary Material. Further inquiries can be directed to the corresponding author.

## Supplementary Materials

The following supporting information can be downloaded at: https://www.mdpi.com/article/10.3390/foods14152728/s1, Figure S1: Chromatogram of components contained in vinegar. (**A**) Chromatogram of free sugar. (**B**) Chromatogram of organic acid. OA, oxalic acid; CA, citric acid; MEA, maleic acid; MA, malic acid; MOA, malonic acid; SA, succinic acid; LC, lactic acid; FA, fumaric acid; AcOH, acetic acid. (**C**) Chromatogram of amino acid.
