# Supplementary material for: Quality Status and Skin-Related Functional Properties of Traditional Korean Fermented Vinegars
Source: Foods. 2025 Aug 4;14(15):2728. doi: 10.3390/foods14152728 (PMC12346884; doi:10.3390/foods14152728)
Supplement: Supplementary file 1 [file foods-14-02728-s001.zip › foods-3733813-supplementary.pdf]

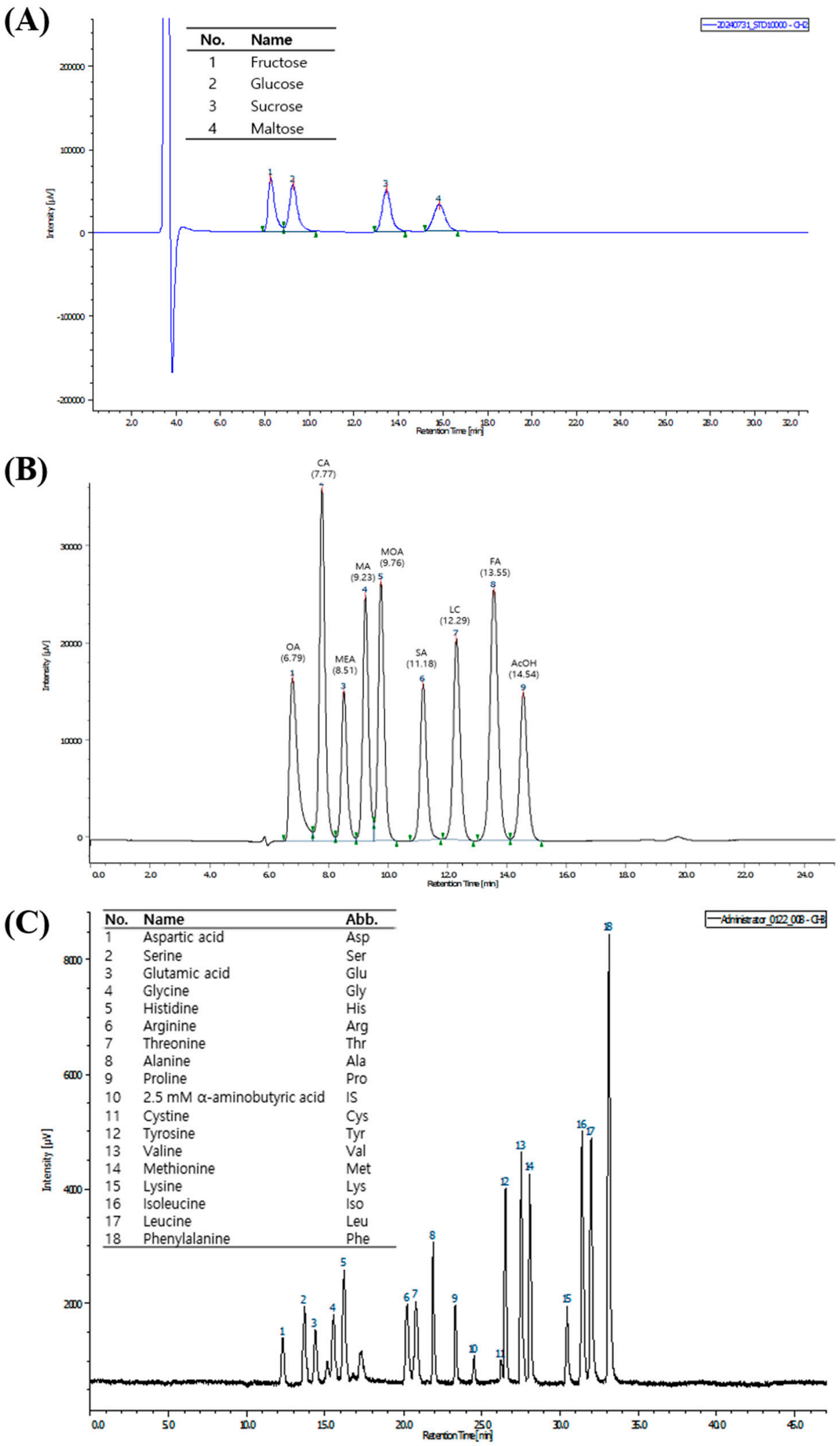

Figure S1. Chromatogram of components contained in vinegar. **(A)** Chromatogram of free sugar. **(B)** chromatogram of organic acid. OA, oxalic acid; CA, citric acid; MEA, maleic acid; MA, malic acid; MOA, malonic acid; SA, succinic acid; LC, lactic acid; FA, fumaric acid; AcOH, acetic acid. **(C)** chromatogram of amino acid.
